# Effect of Cannabis Smoke Condensate on *C. albicans* Growth and Biofilm Formation

**DOI:** 10.3390/microorganisms9112348

**Published:** 2021-11-13

**Authors:** Neftaha Tazi, Xavier Pigeon, Jérôme Mulamba Mbuyi-Boisvert, Simon Giret, François Béland, Mahmoud Rouabhia

**Affiliations:** 1 Groupe de Recherche en Écologie Buccale, Faculté de Médecine Dentaire, Université Laval, Québec, QC G1V 0A6, Canada; neftaha.tazi.1@ulaval.ca; 2 SiliCycle^®^ Inc. 2500, Parc-Technologique Blvd, Québec, QC G1P 4S6, Canada; xavierpigeon@silicycle.com (X.P.); jeromeboisvert@silicycle.com (J.M.M.-B.); SimonGiret@silicycle.com (S.G.); francoisbeland@silicycle.com (F.B.)

**Keywords:** cannabis, *C. albicans*, growth, biofilms, oxidative stress

## Abstract

The most common use of cannabis is smoking. The oral ecosystem, among other constituents, can be deregulated by the presence of cannabis smoke in the oral cavity. We evaluated the effect of cannabis smoke condensate (CSC) on the behavior of *Candida albicans*, a common yeast found in the oral cavity. The yeast was first cultured with different concentrations of CSC, and its growth was evaluated. The transition from the blastospore to the hyphal form and the hyphae size were assessed after 3 and 6 h, along with biofilm formation after 72 h of contact with CSC. The response of *C. albicans* to oxidative (H_2_O_2_) stress was also examined. Our results show that CSC contained high amounts of THC (about 1055 ppm), CBN (63 ppm), and CBG (about 47 ppm). The presence of various concentrations of CSC in the culture medium increased *C. albicans* growth. CSC also contributed to increases in both the hyphal length and biofilm mass. Following oxidative stress (H_2_O_2_ at either 100 or 500 μM), CSC prevented the damaging effect of H_2_O_2_ on both *C. albicans* shape and growth. These findings support clinical observations demonstrating that cannabis may promote *C.* *albicans* growth and oral candidiasis.

## 1. Introduction

The mouth, a critical point of entry to the body, contains different tissue structures, including soft (the gingival mucosa and tongue) and hard (the teeth, bone, and hard palate) tissues. These tissue structures play key roles in oral health. The concept of oral health includes physical, mental, and social well-being [1]. In addition to the different tissues, the mouth (oral cavity) houses a complex microbiota ecosystem consisting of hundreds of bacterial and fungal species. One of the most opportunistic microorganisms in the human oral cavity is *Candida albicans (C. albicans).* As part of the normal microbial flora found in the oral cavity, this ubiquitous yeast is the most common cause of mucosal and invasive fungal infections observed in humans [2,3]. Oral candidiasis can be promoted under a variety of conditions, including the presence of exogenous substances, such as cigarette and cannabis smoke [4,5].

Cannabis is derived from the *Cannabis sativa* plant following drying and pressing [6] and is used all over the world. In 2016, cannabis use was estimated to involve approximately 3.9% of the world’s population aged 15–64 years [7], with the number of cannabis users undoubtedly increasing with liberalized policies governing cannabis possession and use [8]. Cannabis use can take place through smoking, ingestion, or skin absorption, with smoking the dried product in the form of a cigarette representing the easiest and most common use. Cannabis smoke may be associated with different health effects due to the various chemicals it generates [9,10,11]. It has, in fact, been reported that the chemicals emitted from cigarette smoke and cannabis smoke are qualitatively similar, with a few quantitative differences [12,13,14]. More importantly, nitrogen oxides, hydrogen cyanide, and aromatic amines have been found at higher concentrations in cannabis smoke than in cigarette smoke [12]. Furthermore, the total particulate matter (TPM) and ‘tar’ commonly associated with cigarette smoke have been found in similar or higher concentrations in cannabis smoke [12,15]. Cannabis smoke condensates also contain chemical compounds similar to those detected in tobacco cigarette smoke [12,16]. Cannabis smoke has also been shown to have adverse effects on the cardiovascular [17] and respiratory [10] systems and oral health [11].

As the first site of entry, the oral cavity could manifest deregulation following contact with cannabis smoke. Cannabis smokers have been shown to have poorer oral health, with higher decayed, missing, and filled teeth scores; high plaque scores; and less healthy gingiva [18]. Cannabis smokers also suffer from xerostomia [19,20]. Cannabis smoke-related xerostomia could lead to infectious diseases, including oral candidiasis. The effect of cannabis smoke has been shown to deregulate the oral tissue structure [12,21] and the oral ecosystem [4]. An increased prevalence of *C. albicans* in cannabis users has also been shown compared with that in tobacco-smoking scenarios and non-smoking controls [4,19]. We previously demonstrated that cigarette smoke condensate promoted *C. albicans* growth and formed biofilm [22]. This could also apply to cannabis smoke condensate. The purpose of this study was thus to evaluate the effect of cannabis smoke condensate on *C. albicans* growth, the morphological change from blastospores to hyphae, the capacity to form a biofilm, and behavior under oxidative stress (exposure to H_2_O_2_).

## 2. Materials and Methods

### 2.1. Candida Strain

Frozen *C. albicans* (ATCC SC5314) cells were cultured in Sabouraud liquid medium supplemented with 0.1% glucose, pH 5.6. The cultures were performed under a 5% CO_2_ humid atmosphere. After 24 h of incubation, *C. albicans* cells were sub-cultured under the same conditions. The yeast cells were then collected, washed with phosphate-buffered saline (PBS), counted with a hemocytometer, and adjusted to 10^7^/mL prior to use.

### 2.2. Preparation of the Cannabis Condensate

Cannabis cigarettes (dried leaf material) were purchased from the Société Québécoise du Cannabis (SQDC) (Québec City, QC, Canada). Each cigarette had a net weight of 0.5 g and contained 1.7 mg of THC and less than 0.1 mg of CBD, as specified by the manufacturer. To generate the CSC, each cannabis cigarette was inserted into one end of a silicone tube, while the other end of the tube was linked to the smoke chamber consisting of a 12 × 12 cm polypropylene container. A peristaltic pump was used to deliver the cannabis smoke into the exposure chamber. Three cannabis cigarettes were burned, with the smoke collected inside the smoking chamber. Cannabis smoke chemicals adhering to the polypropylene container were extracted using 10 mL of 95% ethanol. This solution was evaporated under a chemical hood to reduce the 10 mL of ethanol to 1 mL. This 1 mL volume of cannabis smoke condensate was then mixed with 59 mL of PBS or Sabouraud medium to constitute a stock solution of cannabis smoke condensate (CSC) at a total volume of 60 mL, representing 20 mL per cannabis cigarette, as we previously reported with cigarette smoke condensate [22,23].

### 2.3. HPLC Analyses

Chemicals and Reagents: All of the chemicals used were of analytical HPLC grade. Water, acetonitrile, methanol, and ethanol were purchased from Fisher Scientific (Saint-Laurent, QC, Canada). Ammonium formate was purchased from Sigma-Aldrich (Oakville, ON, Canada), and formic acid was purchased from Fisher Chemical (Canada). A certified standard mixture of eleven phytocannabinoids, namely cannabidivarin (CBDV), cannabidiolic acid (CBDA), cannabigerolic acid (CBGA), cannabigerol (CBG), cannabidiol (CBD), cannabinol (CBN), tetrahydrocannabinolic acid (THCA-A), Δ^9^-tetrahydrocannabinol (Δ^9^-THC), Δ^8^-tetrahydrocannabinol (Δ^8^-THC), (±)-cannabichromene (CBC), and tetrahydrocannabivarin (THCV), was purchased from Cayman Chemical Co. Inc.

Preparation of Standard Solution: Appropriate aliquots of a standard mixture of phytocannabinoids were diluted with acetonitrile to obtain the following concentrations: 1.25, 2.50, 6.25, 25.0, 50.0, 62.5, and 75.0 µg/mL. These solutions were stored at −20 °C until use.

HPLC Equipment and Chromatographic Conditions: All chromatographic runs were carried out using an Agilent 1260 Infinity II LC system, which consisted of a G7112B binary pump, a G7167A multisampler, a G7116A multicolumn column thermostat, and a G7117C Diode Array Detector, and were recorded at 228 nm. Chromatographic separations were achieved using a Restek Raptor ARC-18 analytical column (4.6 mm × 150 mm × 2.7 µm). The mobile phase consisted of a mixture of acetonitrile and water (75:25) containing 0.1% *v*/*v* formic acid and 5 mM of ammonium formate. Following each run, the HPLC column was washed with a mixture of acetonitrile, ethanol, and water (75:20:5). All analyses were performed using the following conditions: flow rate, 1.5 mL/min; acquisition time, 10.0 min; washing time, 5 min (during the rinse period, the mobile phase composition was 95:5); column temperature, 30 °C; and injection volume, 5.0 μL. All samples and standards were injected at 4 °C.

Sample Preparation: Cannabis smoke condensate solution was diluted 1:25 with a mixture of methanol and water (60:40), then filtered through a 0.22-µm PTFE chromatography syringe filter prior to HPLC analyses. The lower limit of quantification (LLOQ) was 1.25 ppm.

### 2.4. Susceptibility of C. albicans to CSC Conditions

This study used 1, 5, 10, and 20% concentrations of CSC. These concentrations were selected on the basis of previous studies with cigarette smoke condensate at similar concentrations [22,23]. *C. albicans* (10^5^ cells) in 200 μL of Sabouraud liquid medium with or without 1, 5, 10, or 20% CSC were placed in each well of a 96-well plate. The cultures were then incubated at 30 °C in a 5% CO_2_ incubator. Yeast growth was evaluated with the absorbance at 530 nm using an xMark microplate spectrophotometer (Bio-Rad, Mississauga, ON, Canada). The absorbance readings were determined immediately before culture (T0), then every two hours up to 24 h (T24). The experiment was conducted in triplicate and repeated four times. The effect of CSC on *C. albicans* growth was also evaluated using the commercial BacTiter-Glo microbial cell viability assay (Promega, Madison, WI, USA), which measures ATP, an indicator of bacterial viability. Luminescence was quantified using a Synergy 2 microplate reader. The experiment was conducted in triplicate and repeated two times.

### 2.5. Effect of Cannabis Condensate on the Transition of C. albicans from Yeast to Hyphal Form

*C. albicans* cells (10^6^/well) were resuspended in 2 mL of Sabouraud medium in the presence of 10% fetal bovine serum and placed into 6-well plates. The culture medium was then supplemented with CSC at 1, 5, 10, or 20% or not supplemented, and the cultures were incubated thereafter at 37 °C for 6 h. The presence of 10% fetal bovine serum and incubation at 37 °C were considered appropriate conditions to promote hyphal transition [24]. At the end of the 6 h incubation period, the cells were observed under an inverted optical microscope and photographed to document cell morphology and hyphal length. We used Meiji Techno MA285 Microscope Stage Micrometer, 1 mm/0.01 mm scale (Cole-Parmer^®^, Montreal, QC, Canada) to measure the size of each hyphae form. Data were generated from 5 different and independent experiments. Ten photos were taken for each experiment and condition, and the hyphae formed in each photo were analyzed.

### 2.6. Effect of CSC on C. albicans Biofilm Formation

A porous collagen scaffold (Zimmer^®^ Collagen Tape; Zimmer Dental Inc., Carlsbad, CA, USA) was used to provide the yeast cells with an appropriate environment for adhesion and growth. Briefly, the scaffold was cut into small samples approximately 5 × 5 mm in size; each sample was weighed and placed in 12-well plates, with a single piece per well. They were then rinsed twice with Sabouraud medium and seeded with *C. albicans* ATCC-SC5314 (10^5^ cells) in 2 mL of medium with or without 1, 5, 10, or 20% CSC. The samples were incubated for 72 h in a 5% CO_2_ incubator at 37 °C with medium and CSC refreshment every 24 h. In the first set of experiments, biofilm formation was assessed by weighing the collagen membranes at 72 h. The final biofilm weight was obtained by subtracting the initial and final weight measures of each collagen membrane. In the second set of experiments, biofilm formation was assessed by Inspect F50 scanning electron microscopy. In the third set of experiments, *C. albicans* cell viability/metabolic activity in the biofilm was assessed using the MTS (methyl thiazolyl tetrazolium salt) colorimetric assay (ab197010, Abcam, Cambridge, UK). Results are presented as means ± SD (*n* = 4).

### 2.7. Effect of CSC on C. albicans Responses to Stressful Agents

To investigate the effect of CSC on *C. albicans’* sensitivity/resistivity to oxidative stress, cells (10^5^) were cultured in Sabouraud medium with or without H_2_O_2_ (100 or 500 μM) for 60 min at 37 °C in a 5% CO_2_ cell culture incubator. Thereafter, the cells were washed twice with sterile phosphate buffer solution (PBS) to eliminate H_2_O_2_, and the pellets were suspended in 1 mL of fresh Sabouraud medium in the presence or absence of CSC at various concentrations (1, 5, 10, or 20%). From each suspension, 20 μL were placed on Sabouraud agar and incubated at 37 °C for 24 h to assess yeast colony formation. The remainder of each cell suspension was incubated at 37 °C for 24 h. Cell shape was examined by scanning electron microscopy, while cell viability/growth was determined by hemocytometer cell counting (*n* = 4).

### 2.8. Statistical Analyses

Each experiment was performed at least three times, and the experimental values are expressed as means ± SD. The statistical significance of the differences between the control (absence of CSC) and test (presence of CSC) values was determined by two-way ANOVA. Posteriori comparisons were performed using Tukey’s method. Normality and variance assumptions were verified using the Shapiro–Wilk test and the Brown and Forsythe test, respectively. All the assumptions were fulfilled. The *p* values were declared significant when ≤0.05. The data were analyzed using GraphPad INSTAT software, version 3.

## 3. Results

### 3.1. Chemical Characterization of the CSC

As shown in Table 1, the HPLC analyses demonstrated the presence of different chemicals, including Δ^9^-THC, CBN, and CBG. The most prominent chemical present in the CSC was THC, with a concentration of 1055.15 ppm. It should be noted that each cannabis cigarette comprised 0.5 g of dried cannabis leaf and contained 1.7 mg of THC and 0.1 mg of CBD.

### 3.2. Cannabis Smoke Condensate Contributed to C. albicans Growth

**Figure 1:**
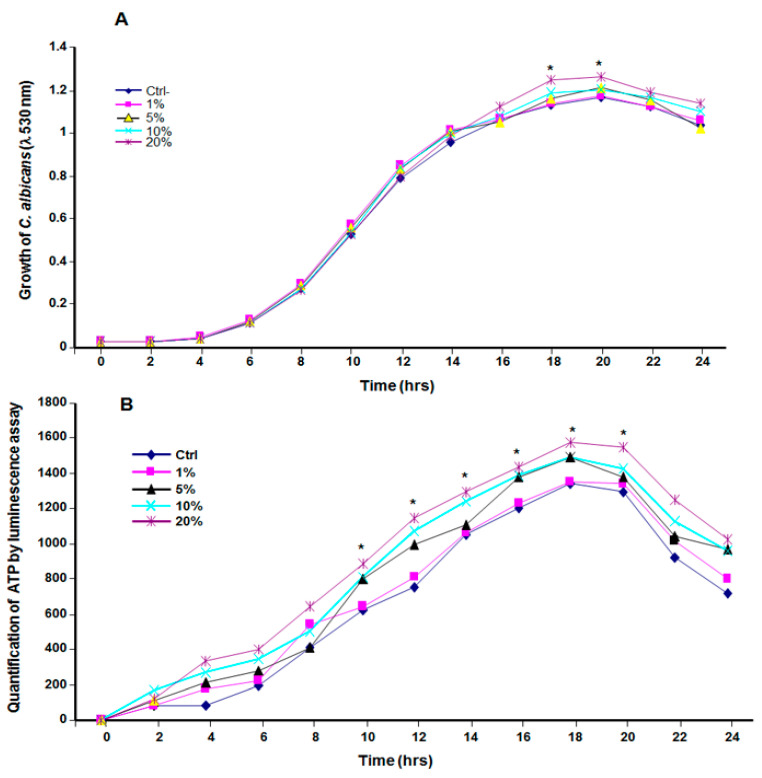
Growth kinetics of *C. albicans* ATCC SC5314 in the presence of different concentrations of CSC. Standardized yeast cell suspensions were exposed to different concentrations (0, 1, 5, 10, and 20%) of CSC and cultured at 30 °C. Growth was measured every 2 h up to 24 h and was plotted (*n* = 4). Panel (**A**): evaluation of the growth using absorbance at λ = 530 nm; panel (**B**): evaluation of cell viability/growth using a commercial luminescence assay (BacTiter-Glo). * *p* ≤ 0.05 when comparing the treated samples with the control.

The exposure of *C. albicans* to CSC led to the increased growth of the yeast (Figure 1). Indeed, starting at 10 h of exposure, *C. albicans* growth in the presence of CSC was higher than that observed in the control (Figure 1B). This growth was maintained to up to 20 h of contact (Figure 1A,B). Both the absorbance measurement and the ATP quantification suggest that *C. albicans* took advantage of the cannabis soluble products to grow.

### 3.3. CSC Increased the Size of C. albicans Hyphae

**Figure 2:**
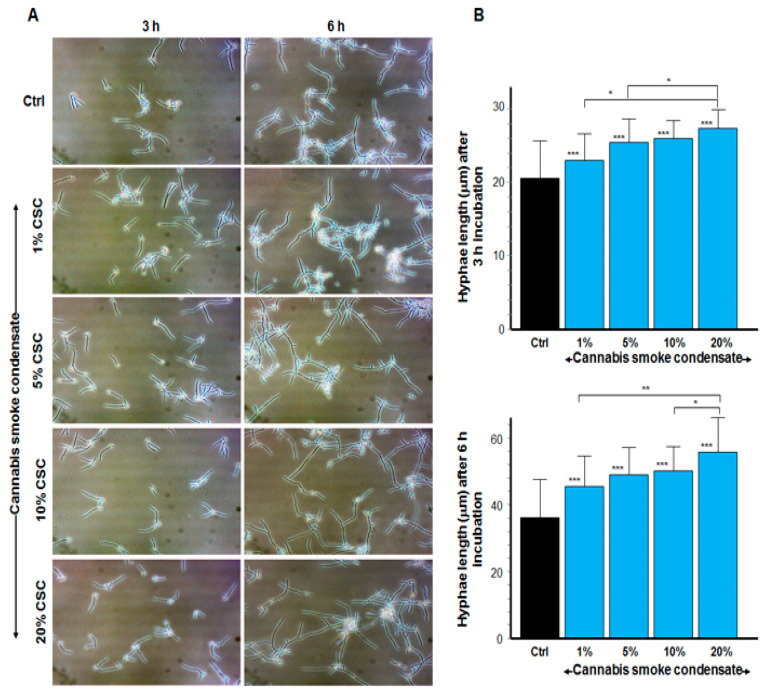
Cannabis smoke condensate increased the length of *C. albicans* hyphae. Cells were cultured in Sabouraud medium supplemented with 10% fetal bovine serum and CSC at various concentrations for 3 and 6 h at 37 °C. Following each culture period, cell shape was observed under an inverted microscope and photographed (**A**). Hyphal tube length was measured using NIH ImageJ software, and the data from 5 separate experiments were analyzed and presented (**B**). Significance for hyphae length was obtained by comparing the CSC-treated and non-treated cultures. * *p* ≤ 0.05; ** *p* ≤ 0.01; *** *p* ≤ 0.001 when comparing the test with the control. Scale bar = 50 μm.

As shown in Figure 2A, the yeast–hyphae transition was observable as early as 3 h of culture under specific hyphal conditions (serum at 37 °C). Indeed, hyphae numbers appeared to be greater with CSC than they were in the control. The transition was observed with every CSC concentration (1, 5, 10, and 20%), and a longer incubation (6 h) led to high-floating hyphal cell aggregates. Hyphae size was measured, showing longer hyphal tubes in the CSC-exposed *C. albicans* cultures than in the control (Figure 2B). Indeed, at 3 h of incubation, the hyphal length was recorded at 20.3 ± 3 μm in the control, 23.5 ± 6.7 μm with 1% CSC, 25.6 ± 3.6 μm with 5%, 26 ± 1.5 μm with 10%, and 27.8 ± 1. 7 μm with 20% CSC. *C. albicans* hyphal forms were longer after 6 h of exposure to CSC than they were after 3 h (Figure 2B). These data show that CSC increased the hyphal length of *C. albicans* compared with the control.

### 3.4. CSC Increased C. albicans Biofilm Formation

**Figure 3:**
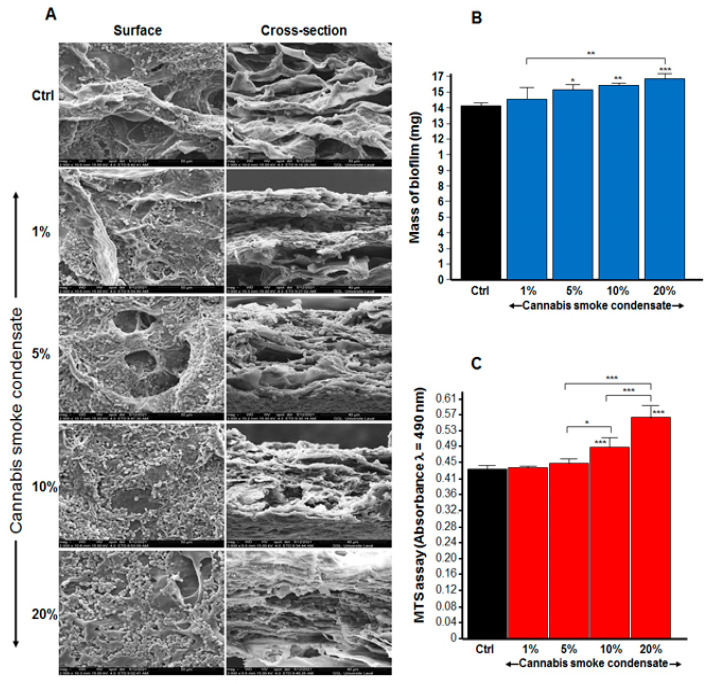
Cannabis smoke condensate increased biofilm mass. *C. albicans* was cultured on a 3D porous collagen scaffold in the presence of CSC with medium and CSC changed every 24 h. After 3 days, *C. albicans* biofilm formation was assessed. Panel (**A**): SEM analyses. Panel (**B**): Mass determinations of the biofilm. Panel (**C**): Cell viability/metabolic activity, as determined by MTS assay. Results are means ± SD (*n* = 4). * *p* ≤ 0.05; ** *p* ≤ 0.01; *** *p* ≤ 0.001.

Because CSC increased *C. albicans* growth, we evaluated its potential effect on *C. albicans* biofilm formation. As shown in Figure 3A, following the surface and the cross-section observations of the scaffold, our SEM analyses revealed high cell density. The increase in biofilm formation after repeated exposure to CSC was particularly elevated with 5, 10, and 20% CSC. To confirm these SEM analyses, we conducted quantitative analyses using dry-weight biomass measurements. Figure 3B shows a significant (*p* < 0.01) increase in the biofilm mass in the presence of 5, 10, and 20% CSC; it should be noted that the greater the CSC concentration, the higher the *C. albicans* biofilm formation. Indeed, the biofilm mass was 14 ± 0.0002 mg in the control, 14.8 ± 0.0009 mg with 1% CSC, 15.5 ± 0.0004 mg with 5%, 15.9 ± 0.0002 mg with 10% CSC, and 16.5 ± 0.0004 mg 20% CSC. This was confirmed by MTS assay (Figure 3C).

### 3.5. CSC Protected C. albicans against Oxidative Stress

**Figure 4:**
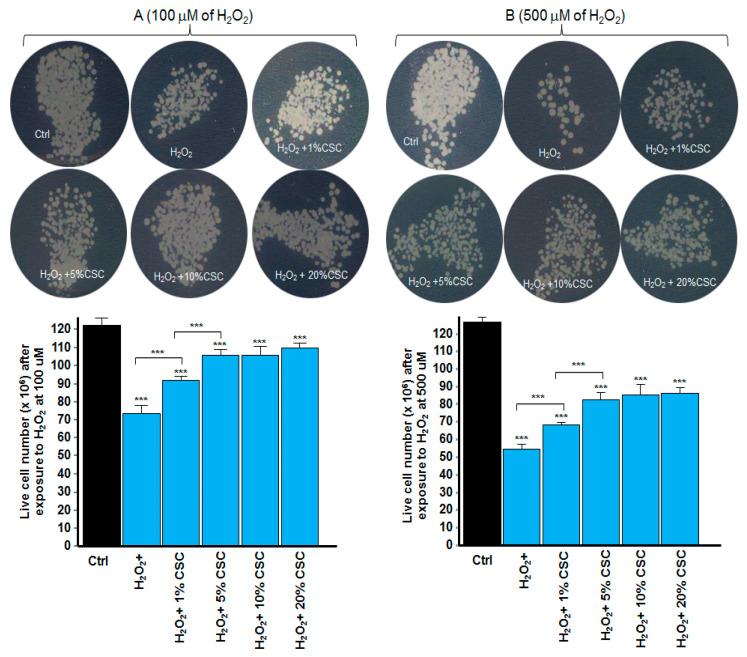
CSC protected the fungi from oxidative stress damage. Cells were incubated for 1 h with or without H_2_O_2_ in the presence or absence of CSC. Panel (**A**): Cell incubation with 100 μM of H_2_O_2_. Panel (**B**): Incubation of the cells with 500 μM of H_2_O_2_. *C. albicans* cells non-exposed to H_2_O_2_ or CSC; *C. albicans* cells exposed to H_2_O_2_ only; *C. albicans* cells exposed to H_2_O_2_ and then to 1% CSC; *C. albicans* cells exposed to H_2_O_2_ and then to 5% CSC; *C. albicans* cells exposed to H_2_O_2_ and then to 10% CSC; and *C. albicans* cells exposed to H_2_O_2_ and then to 20% CSC. Results are means ± SD (*n* = 4). *** *p* ≤ 0.001 compared with the control (no CSC).

**Figure 5:**
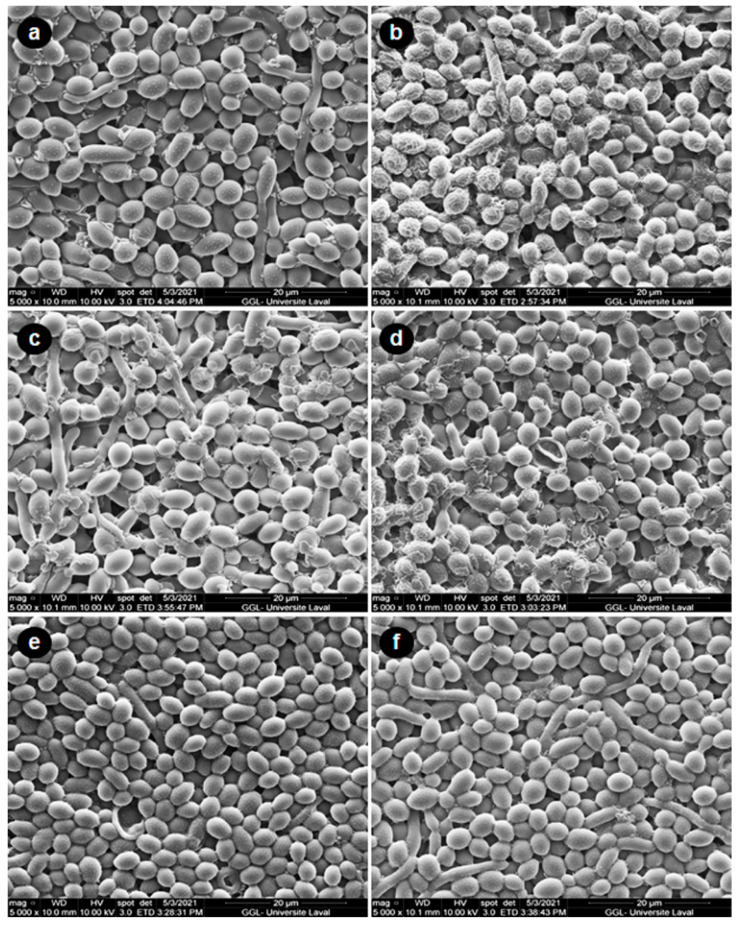
Effect of CSC on *C. albicans* morphology following exposure to oxidative stress. Cells were exposed or not exposed to H_2_O_2_ (500 μM) for 60 min at 30 °C. They were then washed twice and suspended in a culture medium with or without CSC. After 24 h of incubation, the cells were subjected to SEM analyses. (**a**) Control cells; (**b**) cells exposed to H_2_O_2_ alone; (**c**) H_2_O_2_ + 1% CSC; (**d**) H_2_O_2_ + 5% CSC; (**e**) H_2_O_2_ + 10% CSC; and (**f**) H_2_O_2_ + 20% CSC.

As shown in Figure 4A, following exposure to 100 μM of H_2_O_2_ for 60 min, and then culture for 24 h in its absence, there was a significant decrease in the number of colonies (Figure 4A,B) on the surface of the Sabouraud agar medium. This was confirmed by a significant (*p* < 0.001) decrease in the viable cell number compared with that observed with the non-exposed cells. The *C. albicans* culture exposed to 100 μM of H_2_O_2_ in the presence of CSC showed a high number of colonies (c to f). Similar observations were made after exposing *C. albicans* to 500 μM of H_2_O_2_ for 60 min, followed by culture in the presence of CSC for 24 h (Figure 4B). These results were confirmed by the SEM analyses of the cell surface. As shown in Figure 5, *C. albicans* exposed to 500 μM of H_2_O_2_ for 60 min and then cultured for 24 h in its absence displayed a disorganized cell shape with substantial cell membrane wrinkling. After exposure to CSC at 10% (Figure 5e) and 20% (Figure 5f), these features disappeared, resulting in a cell shape comparable to that observed in the control cells (Figure 5a). These results demonstrate that CSC protected *C. albicans* from the oxidative stress induced by H_2_O_2_.

## 4. Discussion

Because the most common method of consuming cannabis is by smoking, and as cannabis smoke comes in direct contact with the buccal environment, we hypothesized that cannabis smoke compounds could interact with oral cavity constituents. The oral ecosystem contains different microorganisms, including *C. albicans*, which may be affected by cannabis products.

In the first set of experiments, we demonstrated that *C. albicans,* an oral opportunist pathogen, took advantage of the presence of CSC in the culture medium to grow better than in the non-exposed control. Although this is the first study demonstrating the direct effect of CSC on *C. albicans* growth, it supports the findings of Darling et al. showing an increased prevalence of candida among cannabis users [4]. The various concentrations of CSC we tested ranged from 1 to 20%, equivalent to 0.025 to 0.5 burned cannabis cigarettes. Such consumption, considered under the recreational use of cannabis, is estimated to be 1.1 g of cannabis flower/leaf per day [25].

*Candida* virulence is limited to extensive cell growth and is promoted by its morphological transition from a spheroid cell shape to hyphal tubes. The hyphal forms were shown to contribute to *C. albicans* invasion of the tissue, causing significant health problems [26,27]. This study demonstrates that supplementation of culture medium with CSC led to an increase in both hyphae density and length. This increased hyphal length could contribute to *C. albicans* tissue invasion [28]. Because the CSC used in this study contained a high level of THC (Table 1), *C. albicans* growth and its yeast-to-hyphae transition may be due to the presence of THC and other chemicals present in the CSC [29,30,31]. Further studies are required to validate this hypothesis and could include purified THC products and their use at various concentrations.

Hyphae have been found to contribute to biofilm formation [32]. Because CSC increased the growth and length of *C. albicans* hyphae, we investigated whether CSC could impact biofilm formation in this yeast and demonstrated that CSC increased the mass of the formed biofilm. Comparable studies using cigarette smoke condensate [22] suggest a common effect between cigarette smoke condensate and cannabis smoke condensate. This effect could be due to the various chemicals found in the CSC. Indeed, in a clinical study showing a prevalence of candida in cannabis users, Darling et al. attributed the increased number and density of *C. albicans* in cannabis smoke to the presence of cannabis hydrocarbons, which may act as an energy source for certain *Candida* species [4]. The present study supports this, as CSC contains all soluble chemicals released following cannabis cigarette burning into an aqueous solution. This solution could thus contain nitrogen oxides, hydrogen cyanide, and aromatic amines, contributing to *C. albicans* growth and biofilm formation [12,15]. The increase in biofilm mass could be due to the presence of hyphal forms following the culture in the presence of CSC. Further studies should examine the specific effect of each chemical we identified in the CSC on *C. albicans* pathogenesis.

As CSC promoted *C. albicans* growth and biofilm formation, CSC could help the yeast escape the stressful situation and continuous growth, leading to oral candidiasis. Indeed, as demonstrated in this study, the exposure of *C. albicans* to H_2_O_2_ (as an oxidative stress situation), followed by incubation in the presence of CSC, helped *C. albicans* to overcome the adverse effect of H_2_O_2_, as evidenced by the increased cell growth. This growth was greater with a higher concentration of CSC. Interestingly, the *C. albicans* cell membrane, which was disorganized because of the contact with H_2_O_2_, displayed a better shape after exposure to the CSC. This suggests that *C. albicans* took advantage of the presence of CSC compounds to grow and to form biofilms, and to prevent adverse effects of a stressful situation.

Further research is required to shed light on the signaling mechanisms adopted by *C. albicans* during exposure to CSC. Our results support a possible link between cannabis use and poor oral health [33,34]. Indeed, cannabis users were found to experience dry mouth immediately after using cannabis, which was maintained for a certain period of time [4,35]. Furthermore, dry mouth was associated with various levels of oral microbial overgrowth, leading to caries and oral candidiasis [36,37]. The use of cannabis leading to dry mouth could thus contribute to *C. albicans* overgrowth and oral candidiasis. It could also be responsible for poor oral health. A recent study showed that more than 30 days of cannabis use was positively associated with multiple periodontal diseases and self-rated fair or poor overall oral health. This study provides evidence that cannabis use is an independent risk factor for poor oral health [38].

## 5. Conclusions

This study demonstrates that cannabis smoke condensate increased *C. albicans* growth and biofilm formation and that cannabis could prevent the adverse effect of oxidative stress after the exposure of *C. albicans* to H_2_O_2_. Our overall data support studies showing more candidiasis in cannabis users.

## Figures and Tables

**Table 1:** Chemicals in the CSC.

Chemical	RT (min)	Concentration (ppm)
CBG	2.98	47.225
CBN	4.75	63.125
Δ^9^-THC	6.03	1055.15

## Data Availability

All data (results) were included in this manuscript.
